# OAGB Bowel Function in Patients With up to 5 Years Follow-Up: Updated Outcomes

**DOI:** 10.1007/s11695-023-06917-4

**Published:** 2023-11-10

**Authors:** Carina Rossoni, Rossela Bragança, Zélia Santos, Octávio Viveiros, Rui Ribeiro

**Affiliations:** 1 Multidisciplinary Center for Obesity Treatment at Hospital Lusíadas, 2724-022 Amadora, Portugal; 2 Institute of Environmental Health (ISAMB), Faculdade de Medicina, Universidade de Lisboa, 1649-026 Lisbon, Portugal; 3 School of Sciences and Health Technologies, Nutrition Sciences, Universidade Lusófona de Humanidades e Tecnologias, 1749-024 Lisbon, Portugal; 4 Nutrition Service of the Centro Hospitalar Univesitário Lisboa Central, 1150-199 Lisbon, Portugal; 5 H&TRC—Health & Technology Research Center, ESTeSL—Escola Superior de Tecnologia da Saúde, Instituto Politécnico de Lisboa, 1990-096 Lisbon, Portugal; 6 General Surgery Department at Hospital Lusíadas Amadora, 2724-022 Amadora, Portugal; 7 Multidisciplinary Center for Obesity Treatment at Hospital Lusíadas Lisboa, 1500-458 Lisbon, Portugal

**Keywords:** One anastomosis gastric bypass, Mini gastric bypass, Bariatric surgery, Metabolic surgery, Bowel function, Nutrition

## Abstract

**Abstract:**

**Objective:**

One-anastomosis gastric bypass (OAGB) is considered an effective technique in weight reduction and remission of comorbidities. However, in common with many bariatric and metabolic/bariatric procedures, gastrointestinal side effects are frequently reported, but clinical experience varies. The objective of this study was to analyze the bowel function of patients who undergo OAGB looking at 5-year postoperative outcomes.

**Method:**

This study is cross-sectional, descriptive and analytical, developed with individuals undergoing OAGB (n = 208) in yhe period between 2015 and 2020. The time periods evaluated were 1 to 6 months (T1), 6 to 12 months (T2), and 1 to 5 years (T3). Data analysis was performed using SPSS v.28.0, considering a significance level *p* ≤ 0.05.

**Results:**

114 participants (54.8%), 79.8% women, mean age 47.0 ± 12.6 years, and BMI 40.1 ± 5.6 kg/m2, 51.9% dyslipidemia, 43.6% arterial hypertension, and 19.1% diabetes mellitus. The T1 group had more severe symptoms/nausea than the T2 group. The T2 group had a significantly lower defecation frequency than the T1 and T3 groups. As for the occurrence of diarrhea, associations were not found in the considered groups. The T3 group had a greater severity of constipation associated with greater difficulty in consuming red meat, white meat, rice, vegetables, and salads.

**Conclusions:**

Gastrointestinal symptoms are prevalent in the first postoperative months. However, diarrhea was not common. The patient selection policy and surgical technique were decisive in this result. Constipation was prevalent in patients between 1 and 5 postoperative years. It was also prevalent in those who had food intolerance, which from a nutritional point of view is an adverse factor for optimal bowel function.

**Graphical Abstract:**

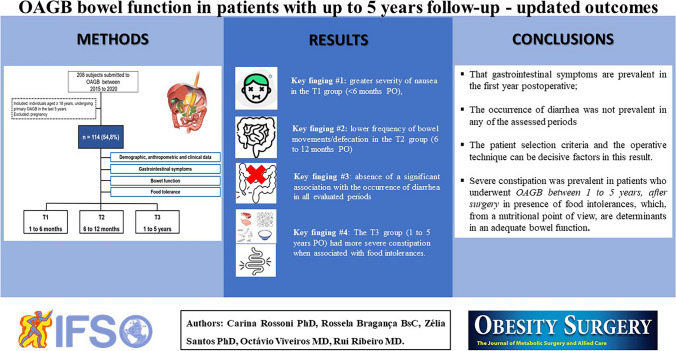

## Introduction

One-anastomosis gastric bypass (OAGB) is the third most performed metabolic/bariatric surgical technique in the world [1]. The popularity of OAGB is due to the greater simplicity of the concept that entails a set of theoretical advantages and, above all, to the quality of its results both in weight reduction and in the remission of comorbidities [2, 3], including type 2 diabetes mellitus [4].

It is considered a hypoabsorptive technique, not free of complications, like all other procedures in the surgical treatment of metabolic diseases. In the literature, the most frequent gastrointestinal complications are gastroesophageal reflux disease, diarrhea, and steatorrhea [5–7].

The occurrence of diarrhea in OAGB is directly related to the exclusion of long intestinal segments (BPL) and, consequently, a reduced absorptive common limb (CL) length, causing pathophysiological changes similar to those of “short bowel syndrome,” with a significant reduction in fat absorption, protein, vitamins, and also hydroelectrolyte imbalance caused by fecal potassium waste and insufficient water absorption [8, 9]. The reduced absorption of fat in the small intestine [10] allows it to reach the colon in greater quantities, creating a cathartic effect.

In addition to these, the inherent anatomical and motility changes in OAGB, with the creation of a blind loop of intestine, can result in small intestinal bacterial overgrowth (SIBO) [11, 12], with potential symptoms such as diarrhea, meteorism/bloating, and abdominal distention [13]. The possible occurrence of pancreatic exocrine insufficiency [14] is another potential cause for steatorrhea and diarrhea.

Post-OAGB gastrointestinal complications require constant surveillance and monitoring since the presence of symptoms greatly compromises the quality of life and health of patients. However, long-term evidence on these digestive alterations, specifically regarding intestinal functioning, are lacking.

The purpose of our study was to analyze the 5 years long-term outcomes in bowel function of patients who underwent OAGB.

## Method

### Study Design and Population

**Fig. 1:**
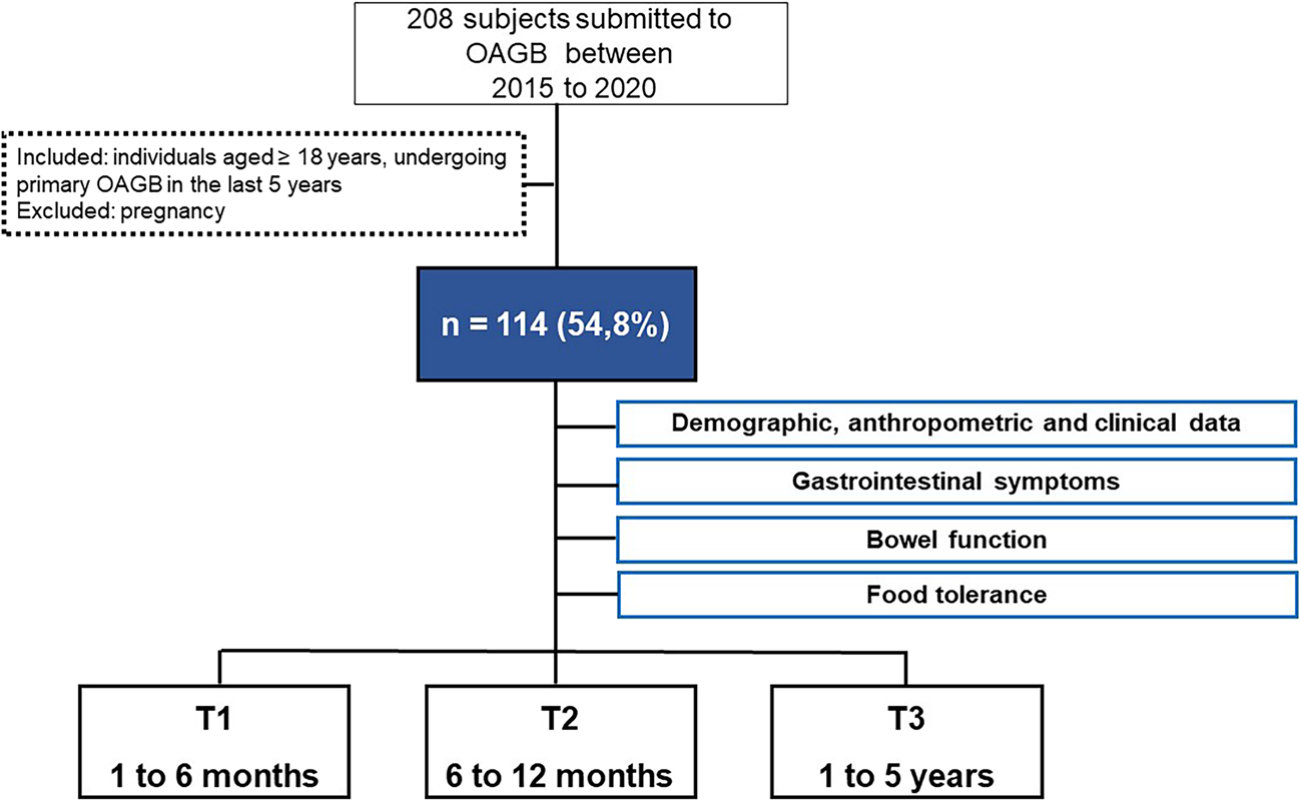
Study design and population

Retrospective cross-sectional, descriptive, and analytical study carried out with individuals undergoing OAGB gastric bypass (*n* = 208) between 2015 and 2020 at an obesity treatment center in Portugal. The sample of this study consists of 114 participants, with the time elapsed since the surgery: 1 to 6 months (T1), 6 to 12 months (T2), and 1 to 5 years (T3). Individuals aged ≥ 18 years, who had undergone primary OAGB in the last 5 years, were included in the study, and cases of pregnancy were excluded (Fig. 1). All participants signed the informed consent form.

This study is part of the multicenter research project entitled: “Gastrointestinal and nutritional parameters in patients undergoing Single Anastomosis Gastric Bypass (SGAB) in different countries – Israel, Spain, Portugal and the United Kingdom,” approved by the Ethics Committee of Hospital Lusíadas Amadora.

### Operative Technique

All participants were evaluated and received medical and nutritional recommendations and others regarding the adoption of good lifestyle habits before and after surgery.

Proton pump inhibitors (pantoprazole 40 mg once daily) were routinely prescribed for at least 1 year, ursodeoxycholic acid (250 mg 3 times a day) for 3 months, and an adapted multivitamin (Fitforme® WLS Primo, 1 caps/day) for life.

The features of the surgical procedure—OAGB—were the following.

**Table:** 

Key steps of the procedure	
Bougie size	36 Fr
Width of pouch	2.5–3 cm
Lower pouch limit	Below *incisura angularis*
Last stapler fire	1–1.5 cm far from GEJ
Length of pouch	15–18 cm
Capacity of pouch	30–40 ml
Biliopancreatic limb length	BMI > 60 kg/m^2^–60% of TBL BMI 50–60 kg/m^2^–50% of TBL BMI 45–50 kg/m^2^–40% of TBL BMI 35–45 kg/m^2^–30% of TBL CL length always longer than 300 cm*
Width of gastroenterostomy	3–4 cm
His angle dissection	Yes
Bowel length measurement	Yes—systematic
Hiatal hernia repair	Yes
Methylene blue test	Yes

*When this criteria results in a CL length less than 3 m, the BPL length is reduced accordingly

### Assessment Tool

Data collection was carried out in follow-up consultations with the multidisciplinary team and through the electronic tool *Survey Monkey*®, in the period between February and December 2020. Data collected were demographic, clinical (comorbidities associated with obesity, type 2 diabetes, hypertension, and dyslipidemia), and anthropometric (weight, height, BMI).

The presence of gastrointestinal symptoms in the 30 days prior to consultation was assessed using the following validated evaluation tools:

#### Gastrointestinal Symptom Rating Scale (GSRS)

This tool consisted of 15 items that assess 5 domains: reflux, indigestion, diarrhea, constipation, and abdominal pain. Each of the questions is evaluated using a seven-grade “Likert”-type scale, where 1 represents no uncomfortable symptoms and 7 represents very uncomfortable symptoms. The highest scores for items and domains represent the presence of more severe symptoms [15–17].

#### Bristol Stool Scale

It’s a tool that evaluates the texture of feces according to seven categories [18–20]:constipation (1 or 2), ideal stool (3 and 4), and diarrhea (5 to 7 ) [21]. Frequency of bowel movements was assessed according to acceptable categories [22].

#### Food Tolerance

Questionnaire based on subjective food satisfaction and tolerance to different foods and frequency of vomiting and regurgitation after bariatric surgery, developed and validated by Suter M. & cols (2007) [23]. The possible answers regarding subjective food satisfaction are very poor, poor, acceptable, good, and excellent. Scores are given depending on the answer: 1 point for very poor and 5 for excellent. Tolerance for red meat, white meat, salad, vegetables, bread, rice, pasta, and fish is individually rated—if the intake of each of these foods occurs easily (2 points), with some difficulties (1 point), or impossible (0 points). In addition, vomiting and regurgitation are scored—if it never occurs (6 points), if it rarely occurs (4 points), and if it frequently occurs (2 points). A score is obtained, with 1 point being the worst possible tolerance and 27 points being the best possible tolerance—perfect [23, 24].

### Statistical Analysis

Quantitative variables were described as mean and standard deviation and ordinal variables as median and interquartile range. Categorical variables were described by absolute and relative frequencies. To compare means, the Analysis of Variance (ANOVA) complemented with post hoc Tukey’s test was applied. In the case of asymmetry, the Kruskal-Wallis test complemented by Dunn’s post hoc test was used. When comparing proportions, Pearson’s chi-square test was applied. The association between ordinal variables was assessed using Spearman’s correlation coefficient. The significance level adopted was 5% (*p* ≤ 0.05), and the analysis was performed using the SPSS program version 28.0.

## Results

There were 114 participants with available follow up data that met the inclusion criteria: 79.8% women, medium age 47.0 ± 12.6 years, and BMI 40.1 ± 5.6 kg/m^2^. The prevalent associated diseases in this population were 51.9% dyslipidemia, 43.6% hypertension, and 19.1% diabetes mellitus. Preoperatively, group T1 (< 6 months post-op) had significantly lower BMI than groups T2 (6 to 12 months post-op) and T3 (1 to 5 years post-op). In addition to this, dyslipidemia was significantly lower at T1 (< 6 months post-op), according to Table 1.

**Table 1:** Demographic, anthropometric and clinical characteristics in patients who underwent OAGB (*n* = 114) up to 5 postoperative years

Characteristics	All groups ± SD or *n* (%)	T1 < 6M PO *n* = 35 (30.7%)	T2 ≥ 6 - 12M PO *n* = 21 (18.4%)	T3 ≥ 1 - 5Y PO *n* = 58 (50.9%))	*p*
Age (years)	47.0 ± 12.6	48.4 ± 10.2	44.2 ± 13.0	47.3 ± 13.7	0.486
Gender					0.557
Female	91 (79.8)	26 (74.3)	18 (85.7)	47 (81.0)	
Male	23 (20.2)	9 (25.7)	3 (14.3)	11 (19.0)	
Antropometrics					
Weight (kg)	111.9 ± 20.1	105.7 ± 15.6^a^	120,9 ± 21.5^b^	112.4 ± 21.0^ab^	**0.026**
Height (m)	1.67 ± 0.09	1.68 ± 0.09	1.67 ± 0.07	1.66 ± 0.09	0.500
BMI (kg∕m^2^)	40.1 ± 5.6	37.3 ± 3.3^a^	43.1 ± 6.4^b^	40.8 ± 5.8^b^	**< 0.001**
Comorbidities					
Hypertension	48 (43.6)	12 (36.4)	9 (45.0)	27 (47.4)	0.592
Dyslipidemia	56 (51.9)	9 (27.3)	13 (68.4)	34 (60.7)	**0.003**
T2 diabetes	*21 (19.1)*	6 (18.2)	6 (18.2)	10 (17.5)	0.757

^a,b^Equal letters do not differ by Tukey’s test at 5% significance

The T1 group (< 6 months PO) had greater severity of nausea symptoms than the T2 group (6 to 12 months PO) (Table 2). Regarding the other gastrointestinal symptoms, there was no significant difference among the groups.

**Table 2:** Occurrence of gastrointestinal symptoms (GRSS Scale) in patients who underwent OAGB (*n* = 114) up to 5 postoperative years

Dimensions	Median (25th–75th percentile)*	T1 < 6M PO *n* = 35 (30.7%)	T2 ≥ 6–12M PO *n* = 21(18.4%)	T3 ≥ 1–5Y PO *n* = 58 (50.9%)	*p*
Pain or discomfort in your upper abdomen or stomach	2 (1–3)	2 (1–2.5)	1 (1–2)	1.5 (1–3)	0.106
Heartburn	1 (1–2)	1 (1–1)	1 (1–2)	1 (1–2)	0.729
Acid reflux	2 (2–2)	2 (2–2)	2 (2–2)	2 (2–2)	0.763
Hunger pains in your stomach	1 (1–2)	1 (1–2)	1.5 (1–2)	1 (1–3)	0.378
Nausea	1 (1–2)	2 (1–3)^b^	1 (1–2)^ab^	1 (1–2)^a^	**0.041**
Noises in your stomach	2 (1.5–3)	2 (2–3)	2 (1–3)	2 (1.5–3)	0.531
Felt your stomach swelling	2 (1–3)	2 (1–2)	2 (1–2)	2 (1–3.5)	0.588
Eructations/burps	2 (1–3)	2 (1.5–3)	2 (1–3)	2 (1–3)	0.382
Flatus	3 (2–4)	3 (2–4)	3 (2–4)	3 (2–4)	0.696
Constipation	2 (1–3)	2 (1–3.5)	1 (1–2)	1 (1–3)	0.129
Diarrhea	1 (1–3)	1 (1–3)	1 (1–2)	2 (1–3)	0.390
Loose stools	2 (1–3)	2 (1–3)	2 (1–2)	2 (1–3)	0.242
Total score	2 (1.6–2.4)	2.1 (1.7–2.4)	1.9 (1.5–2.2)	2.0 (1.6–2.6)	0.298

*Being from 1 to 7 (without the very severe discomfort)

^a,b^Shows post hoc inter group comparisons which did not differ by Dunn’s test at the 5% significance level

Group T2 (6 to 12 months post-op) had significantly lower frequency of bowel movements than groups T1 (< 6 months post-op) and T3 (1 to 5 years post-op), in line with the results described in Table 3.

**Table 3:** Bowel function (Bristol Tools Scale) in patients who underwent OAGB (*n* = 114) up to 5 postoperative years

Dimension	Median (25th–75th percentile) or *n* (%)	T1 < 6M PO *n* = 35 (30.7%)	T2 ≥ 6–12M PO *n* = 21 (18.4%)	T3 ≥ 1–5 Y PO *n* = 58 (50.9%)	*p*
Hard stools	1 (1–2)	1 (1–2.5)	1 (1–3)	1 (1–2)	0.823
Urgent episodes to evacuate	2 (1–3)	2 (1–3)	1 (1–4)	2 (1–3)	0.972
Frequency of bowel movements	2 (1–3)	2 (1.5–4)^b^	1 (1–2) ^a^	2 (2–3)^b^	**0.005**
Frequency of your defecations					0.451
Once in 1–2 days	28 (26.4)	7 (21.2)	5 (26.3)	16 (29.6)	
1–2 times a day	50 (47.2)	14 (42.4)	11 (57.9)	25 (46.3)	
2–3 times a day	13 (12.3)	5 (15.2)	2 (10.5)	6 (11.1)	
3 times a week or less	13 (12.3)	7 (21.2)	0 (0.0)	6 (11.1)	
More than 3 times a day	2 (1.9)	0 (0.0)	1 (5.3)	1 (1.9)	
Stool texture					0.495
Type 1	3 (2.8)	2 (6.1)	1 (5.3)	0 (0.0)	
Type 2	7 (6.6)	2 (6.1)	3 (15.8)	2 (3.7)	
Type 3	20 (18.9)	5 (15.2)	3 (15.8)	12 (22.2)	
Type 4	28 (26.4)	10 (30.3)	3 (15.8)	15 (27.8)	
Type 5	21 (19.8)	6 (18.2)	6 (31.6)	9 (16.7)	
Type 6	26 (24.5)	8 (24.2)	3 (15.8)	15 (27.8)	
Type 7	1 (0.9)	0 (0.0)	0 (0.0)	1 (1.9)	

^a,b^Shows post hoc inter group comparisons which did not differ by Dunn’s test at the 5% significance level

As for the occurrence of diarrhea, associations were not significant in the studied groups. However, the T3 group (1 to 5 years post-op) was the one that presented the greatest severity of constipation associated with greater difficulty in consuming red meat, white meat, rice, legumes, and salads (Table 4).

**Table 4:** Bowel function versus food tolerance in patients who underwent OAGB (n=114) up to 5 postoperative years

Foods	Population *n* = 114 (100%)		T1 < 6 M PO *n* = 35 (30.7%)		T2 ≥ 6–12 M PO *n* = 21 (18.4%)		T3 1–5 Y PO *n* = 58 (50.9%)	
	Diarrhea	Constipation	Diarrhea	Constipation	Diarrhea	Constipation	Diarrhea	Constipation
Red meat	−0.008	0.181	−0.050	−0.111	0.258	0.007	−0.023	0.338*
White meat	0.084	0.351***	0.030	0.217	0.357	0.174	0.102	0.466***
Salad	−0.094	0.254**	−0.261	0.257	−0.308	−0.288	0.150	0.302*
Vegetables	−0.069	0.063	0.000	0.226	−0.308	−0.288	0.038	0.067
Bread	0.147	0.133	0.309	0.074	−0.131	0.069	0.231	0.156
Rice	−0.039	0.240*	0.073	0.247	0.317	−0.201	−0.157	0.329*
Pasta	−0.005	0.172	0.119	0.248	0.155	−0.364	−0.089	0.257
Fish	−0.129	0.191	−0.206	0.090	0.141	0.247	−0.098	0.259
Leguminous	0.050	0.217*	−0.077	0.200	0.233	−0.364	0.128	0.412**

Spearman’s correlation coefficient significance *p < 0.05; **p < 0.01; ***p < 0.001

## Discussion

The main findings of our study were the greater severity of nausea in the T1 group (< 6 months PO), lower frequency of bowel movements/defecation in the T2 group (6 to 12 months PO), and the absence of a significant association with the occurrence of diarrhea in all evaluated periods. However, patients in the T3 group (1 to 5 years PO) had more severe constipation when associated with food intolerance.

Liagre A. et al. [25] identified in their cohort common gastrointestinal symptoms attributed to the anatomical characteristics and disabsorptive effect of OAGB, such as nausea, vomiting, reflux, food intolerances, steatorrhea, and diarrhea.

The prevalence of diarrhea and steatorrhea is one of the major concerns regarding adverse effects after OAGB, especially in procedures with BPL > 200 cm, thus characterizing an important effect of malabsorption [7]. Therefore, measuring the size of the intestine is extremely important, since the definition of BPL and CL lengths has an impact, in addition to metabolic aspects, on the prevention of complications such as diarrhea. Diarrhea, in turn, greatly compromises the quality of life of patients, beyond the implicit risk of malnutrition.

Unlike our results, Zarshenas N. et al. [26] carried out a retrospective cohort study, comparing, among other variables, self-reported gastrointestinal symptoms in patients undergoing OAGB and Roux-en-Y gastric bypass (RYGB), up to 2 years after surgery, noting the presence of diarrhea and steatorrhea in those undergoing OAGB, the latter with a slightly wider gastric tube (40F bougie) and longer BPL, greater than 200 cm. In our study, the protocol used by surgeons to determine the length of the loops is defined by a percentage of the bowel length, defined by the patient’s BMI. In our study, a 36F gauge “bougie” was used, and the average BMI was 40.1 kg/m^2^; therefore, the most used length for the BP loop was 30% of the TBL. Knowing that the average length of the intestine, which we found in our center, is 6.5 m, the length of the BPL was, in theory, around 195 cm.

Nowadays, we know that the longer the BPL in OAGB, the greater its ability to absorb bile acids, which seems to be related to better weight loss. This results in a better control of type 2 diabetes when compared with both the “Sleeve” and the RYGB, these effects being more marked the longer the BPL is [27].

On the other hand, studies with RYGB demonstrated that a longer BPL produces a more intense ileal stimulation and directly proportional to the length of that loop, a fact demonstrated in the proportional increase of GLP1 measured in the portal and systemic venous territories [28]. However, a longer BPL can also produce hypoabsorption and chronic diarrhea with a risk of malnutrition. This varies depending on individual factors (TBL, alimentary and nutritional habits, previous food intolerance—lactose, and allergies—gluten) and populational (genetic and behavioral) factors. Especially in populations with lower protein consumption, for example, in those with predominantly vegetarian habits, a BPL of 250 cm in OAGBs is shown to be significantly (4x) more inducing of hypoabsorption of macro and micronutrients than a BPL of 150 cm, normally in course with diarrhea [29].

The effects of SIBO and exocrine pancreatic insufficiency may partially explain gastrointestinal symptoms and nutritional deficiencies after OAGB, including decreased food intake [13]. Data presented in the study developed by Kaniel O et al. [12] demonstrate that those who developed SIBO have a significantly lower food intake at 6 months after surgery. However, they did not obtain any difference in gastrointestinal symptoms and neither in anthropometric parameters among the groups in the same period.

Other factors such as reduced absorption at the intestinal mucosal surface, reduced production of enteric enzymes (lactase deficiency), and an irritable bowel due to undigested carbohydrates may also play a role in triggering diarrhea [30, 31].

All these factors are important in the therapeutic decision of patients with metabolic disease, and their presence must be carefully considered before opting to perform an OAGB.

In our study, there was no significant increase in the incidence of diarrhea in any of the three periods evaluated, which we attribute to our patients selection policy. Patients with frequent diarrhea, whether due to food intolerances, exocrine pancreatic insufficiency, or other causes, patients who are vegetarians, or those with social conditions that allow us to doubt their ability to obtain a sufficient protein intake, or comply with vitamin-mineral supplementation, are selected for techniques other than OAGB. Our operative technique, through the systematic measurement of the entire small bowel and the rule for deciding the length of the BPL, as well as the criterion of always having a minimum absorptive efferent loop of a minimum of 3 m, very likely contributed to the observed result.

Among the various gastrointestinal symptoms, only nausea was more frequent in the first 6 months after surgery, but without the occurrence of SIBO, which is probably due to reduced food intake due to moderate restriction and the sudden increase of incretin production in this period after OAGB. The probable reason why group T1 (< 6 months) presents more intense nausea than group T2 (≥ 6 to 12 months) is due to the important stimulus of incretins (GLP-1, PYY, etc.) [32, 33], initial gastroparesis that regresses over time. Al-Rasheid N. et al. [34] found in their study that symptoms of persistent nausea and vomiting after surgery were mediated by elevated fasting GLP-1 levels. We can also infer, as a hypothesis, that nausea may be related to the rapid entry of the food bolus into the CL and, consequently, to the sudden distension of the intestinal wall. In a small percentage of cases of bending or torsion of the single anastomosis, the food bolus can preferably be directed toward the BPL, and the resulting distension can also contribute to this aspect [34]. We may also put the hypothesis that in some cases peristaltic reaction may push the food back to the pouch contributing also to nausea and gastro-esophageal reflux as well. Over time, bowel and anastomosis dilation can soften or nullify these mechanisms within months.

It is also necessary to consider the presence of mild dehydration as one of the possible causes.

With respect to lower frequency of bowel movements/defecation in the T2 group than the T1 and T3 groups, it is necessary to highlight that there is no pre-established frequency of defecation; most people will have a bowel movement between three times a day and three times a week; that is, in any of the postoperative phases, it can be considered normal. However, Afshar et al. [35] reported a statistically significant reduction in bowel movements from 8.6 to 5.7 movements per week (*p* = 0.125) and an increase in constipation from 8 to 27% at 6 months after bariatric and metabolic surgery. Defecation frequency can be influenced by diet, hydration, stress, age, medication use, and social circumstances. We believe that the reduced frequency of defecation in the T2 group (≥ 6 to 12 months) compared to the others is related to reduction in total food intake and relative dehydration, highlighting the possible reduction in the intake of dietary fiber and foods with prebiotic action. This phase is expected to be absolutely completed only 2 years after bariatric surgery with adequate, usual, and stable food intake [36]^.^

In the remaining GI symptoms, likewise, there were no differences in the three evaluated periods. Reduced food intake may also be related to food restrictions and/or intolerances, which directly reflects on the inadequacy of the diet (energy, macro- and micronutrients, fiber, and water) and consequently on intestinal functioning. These difficulties were also reported in the study developed by Silva H. et al. [37], when evaluating patients undergoing bariatric and metabolic surgery up to 18 months postoperatively.

The severity of constipation presented by the T3 group (≥ 1 to 5 years) is again associated with greater difficulty in eating red and white meat, rice, vegetables, and salads; we mean poor nutritional quality. Sherif-Dagan S et al. [38] evaluated food tolerance in a multicenter study with people undergoing OAGB. It demonstrated the presence of food intolerances in the short-term postoperative period, but better food tolerance in those with longer follow-up time, up to 5 years. Specifically, food intolerance to rice, fruits, and vegetables is a factor that significantly contributes to the reduction in the intake of carbohydrates and directly fiber [36, 39–41].

Regarding fiber intake, studies carried out 2 years after surgery have demonstrated that fiber intake remains significantly lower than adequate intake, ranging from 10.4 to 11.7 g/day. It is well known this intake is reduced in the first year after surgery [36, 41]. Grosse et al. [41] found that this reduction in fiber intake is present regardless of the surgical technique or postoperative time. In addition to low fiber intake, there is an initial discomfort in the digestive process and dietary restrictions, which also directly influence proper bowel function. It is a worth note to stress that intestinal malfunction, and/or dysfunction, is very common in populations with severe obesity even in the preoperative period [42, 43] and reduced fiber intake may be the cause of the increase in intestinal transit time in the postoperative period. The combination of insoluble and soluble fibers would be recommended, the first having a significant laxative effect and the second an increase in the volume of stools, thus biomass and fermentation by-products, respectively [44].

Although we have not assessed the intake of fiber/day, water/day, and the frequency of physical activity, we interpreted the occurrence of severe constipation in our patients in group T3, submitted to OAGB between 1 and 5 years after surgery, as a consequence of intolerance presented, through the difficulty of ingestion of foods that are good sources of protein, carbohydrates, and fiber.

Constipation, according to the literature, is a characteristic of intestinal functioning in those submitted to the RYGB, sleeve, and adjustable gastric banding techniques [42]. In the OAGB, the occurrence of frequent diarrhea is more commonly described, which did not happen in our study, with constipation being the most frequent, the main finding of this study.

Our study has limitations inherent to its retrospective design and the fact that it represents the experience of a single center.

The high participation of patients, the performance of surgeries by a single surgeon, and the consistent and careful selection of patients are credibility factors for our study. In addition, the surgical protocol that includes the systematic measurement of bowel length and the configuration of the procedure based on the preoperative BMI as explained above, always maintaining an efferent loop with a length greater than 3 meters, give consistency to the findings obtained.

This study may contribute to a better understanding of the evidence on the safety and efficacy of OAGB, as well as to the systematic nutritional management of adverse effects, such as gastrointestinal symptoms, specifically intestinal functioning.

It is still necessary to develop prospective studies that correlate gastrointestinal symptoms with food intolerances, intake of soluble and insoluble dietary fibers, water, and even with the practice of physical exercise in patients undergoing OAGB, also analyzing the relationship with the length of the biliopancreatic and absorptive efferent loops.

## Conclusion

We conclude that gastrointestinal symptoms are prevalent in the first postoperative months; however, the occurrence of diarrhea was not prevalent in any of the assessed periods. The patients’ selection policy and the operative technique can be decisive factors in this result.

Severe constipation was prevalent in patients who underwent OAGB between 1 to 5 years after surgery and who had food intolerance, which, from a nutritional point of view, are important determinants in an adequate bowel function.

## Data Availability

The authors declare to make the data available for future studies.
